# What you plant may not be what you bought: morphological and genetic discordance in specialty *Coffea arabica* L. cultivars from Ecuador

**DOI:** 10.3389/fpls.2026.1868034

**Published:** 2026-07-14

**Authors:** María Fernanda Tapia-Armijos, Elizabeth Gusmán-Montalván, Diego P. Vélez‐Mora, Carlos Iñiguez-Armijos

**Affiliations:** 1 Departamento de Ciencias Biológicas y Agropecuarias, Universidad Técnica Particular de Loja, Loja, Ecuador; 2 VINKA Coffee Farm and Lab, Loja, Ecuador

**Keywords:** DNA fingerprinting, Ethiopian landrace, Gesha cultivar, morphological traits, seed certification, Sidra cultivar, varietal misidentification

## Abstract

The genetic identity of coffee cultivars is fundamental to the specialty coffee sector, where premium prices are paid under the assumption that the purchased planting material corresponds to the declared variety. However, many producing countries lack the certification infrastructure necessary to guarantee this identity in their informal seed systems, exposing producers to undetected varietal non-conformity. In this study, we examine a case from a specialty coffee (*Coffea arabica* L.) farm in southern Ecuador where seeds labeled as Sidra (USD 100/kg) and Gesha (USD 500/kg) were purchased without genetic or phytosanitary certification. Using a combination of SSR-based DNA fingerprinting and quantitative morphological characterization, including plant architecture, leaf functional traits, and fruit characteristics, we documented varietal identity and assessed the discriminant capacity of morphological traits across the four resulting morphotypes. Using eleven microsatellite markers for SSR fingerprinting, we found that two of the four morphotypes did not match their declared commercial identity. One plant sold as Sidra was identified as compatible with Batian, a composite variety of Kenyan origin that is genetically unrelated to Ethiopian landraces. The plants acquired as Gesha corresponded to a pure Ethiopian landrace that is genetically similar to, but not identical to, the Panamanian Geisha reference accession T.02722. Only two morphotypes were confirmed as Sidra. Furthermore, the placement of Sidra within the Core Ethiopia genetic group is consistent with prior population-level analyses and with its likely status as a selected Ethiopian landrace rather than a variety of hybrid origin. Morphological linear discriminant analysis achieved 82.4% overall classification accuracy under leave-one-out cross-validation (LOOCV), with internode length dominating the first discriminant function (LD1 = 66.6%). These results demonstrate that varietal nonconformity in the specialty coffee seed sector can extend to the inadvertent introduction of genetically unrelated material and underscore the urgent need for accessible seed certification.

## 1 Introduction

The expansion of the specialty coffee market has intensified demand for cultivars with well-defined sensory profiles, high productivity, and resistance to disease (Barbosa et al., 2020; Montagnon et al., 2025b). Farmers investing in premium varieties do so under the assumption that acquired planting material faithfully corresponds to its declared genetic identity (Pruvot-Woehl et al., 2020; de Andrade Silva et al., 2023). However, this assumption is frequently incorrect. A recent technical assessment of the quality of *Coffea arabica* seed sector across five Latin American countries found that over 36% of participating seed lots exhibited very high rates of genetic non-compliance. Only 50% or fewer of the tested plants were correctly identified (World Coffee Research, 2024). Global-scale SSR fingerprinting studies have documented similar patterns of non-conformity, even in well-characterized varieties such as Gesha (Pruvot-Woehl et al., 2020; Montagnon and Serito, 2024). This problem is rooted in the absence of regulated seed systems capable of ensuring the identification, certification, and traceability of planting material (Fabian, 2023; Montagnon and Serito, 2024; Petrillo et al., 2025). In their place, informal exchange networks and inadequate verification practices have normalized cultivar misidentification and can introduce material from genetically unrelated varieties, not merely within-cultivar variation (Montagnon and Serito, 2024).

Ecuador exemplifies this broader pattern. Producers of *C*. *arabica*, particularly those cultivating high-value specialty varieties, routinely acquire seeds or seedlings without genetic or phytosanitary certification. The economic consequences extend well beyond the initial purchase price. Given *C. arabica*’s multi-year production cycle, which typically requires three to four years to reach significant harvest levels, investments in planting, canopy management, and variety-specific marketing accumulate under the assumption that the acquired material will express the expected genetic identity. When this does not occur, the economic and reputational burden falls disproportionately on the producer, even when the grower is primarily a victim of a flawed and unregulated supply chain (Perez et al., 2023; World Coffee Research, 2024). While previous studies have documented misidentification on a global or multi-country scale (Pruvot-Woehl et al., 2020; Montagnon and Serito, 2024; World Coffee Research, 2024), farm-level evidence remains absent. Furthermore, the ability of quantitative architectural traits to discriminate between cultivars has not been formally evaluated for the specialty cultivars most at risk, as opposed to simple visual descriptors. This study addresses both gaps, combining SSR-based genetic fingerprinting with standardized morphological characterization to document a concrete case of varietal non-conformity in southern Ecuador and to establish a preliminary morphological reference framework for the Sidra cultivar, for which no verified standard currently exists.

Two varieties illustrate this tension and define the interpretive framework of this study. The Sidra cultivar commands premium prices on international specialty markets for its distinctive cup profile, but its genetic origin has not been formally established, and its commercial identity is unstable, as in many other Ethiopian landrace cultivars (Montagnon and Serito, 2024). Available evidence suggests that material traded as ‘Sidra’ may encompass multiple distinct accessions under the same commercial name, without a fixed reference genotype (Montagnon et al., 2025b). Prior genetic-level analyses place Sidra within the Core Ethiopia genetic group (MontagnonRD2 Vision, 2023), and we treat this affiliation as a testable expectation against which the commercially acquired material is evaluated here, rather than as a pre-confirmed attribute of the purchased seeds. The absence of a fixed genetic or morphological reference for Sidra complicates authentication, hinders formal variety protection, and limits the traceability claims that premium markets increasingly demand. On the other hand, the Gesha cultivar is well characterized, with its origin traceable to collections made in the Gori Gesha forest in southwestern Ethiopia (Medrano et al., 2025) and a verifiable genetic fingerprint linked to the Panamanian lineage specifically descended from CATIE accession T.2722 (World Coffee Research, 2023a). This clear genetic identity provides an established authentication anchor yet mislabeling and contamination during seed multiplication can still affect its genetic purity in commercial channels (Pruvot-Woehl et al., 2020). These contrasting authentication profiles — Sidra without a fixed reference, Gesha with one — generate qualitatively different traceability risks and define the interpretive context for evaluating both the genetic results and their commercial implications reported here. SSR microsatellite markers are well suited to resolving both cases, offering the resolution necessary to distinguish closely related genotypes that morphological assessment alone cannot reliably separate (Vieira et al., 2010; Sousa et al., 2017).

Morphological characterization is a practical and field-accessible complement to SSR-based identity verification because plant architectural traits, particularly branch internode length, have been shown to discriminate between *C. arabica* cultivars (Matsunaga et al., 2016; Rakočević, 2025). These traits reflect genotype-dependent patterns of meristematic elongation that are more sensitive to environmental conditions than leaf-level functional traits (Martin et al., 2017; Scalabrin et al., 2020; Salojärvi et al., 2024; Montagnon et al., 2025a). However, whether morphological variation reliably tracks genetic differentiation among specialty cultivars like Sidra and Gesha remains an open question requiring further empirical study. This question is especially important for Sidra because it lacks a fixed genetic reference profile and a verified set of morphological descriptors. This dual absence makes field-based authentication difficult, complicates formal variety protection, and limits the phenotypic standardization that premium markets increasingly require for traceability protocols. Integrating SSR fingerprinting with standardized architectural trait characterization therefore offers a practical framework for cultivar verification that could begin to address this gap.

Here, we report on a case study from a specialty coffee farm in Loja, southern Ecuador, where seeds commercially labeled as Sidra and Gesha were acquired at premium prices (USD 100 kg and USD 500 kg, respectively) without official certification. Unexpected morphological variation observed in the field prompted a formal evaluation combining SSR-based genotyping with quantitative morphological assessment of four resulting morphotypes. Our objectives were to: 1) verify the genetic identity of each morphotype using SSR fingerprinting against a validated reference database; 2) quantify morphological variation among morphotypes using standardized plant architectural, leaf functional, and fruit traits; 3) evaluate the discriminant capacity of morphological traits relative to SSR-based genetic identity; and 4) contextualize these findings within the broader challenges of seed traceability and producer vulnerability in regions lacking formal certification systems. Based on the degree of phenotypic divergence observed among morphotypes and on prior evidence that morphologically distinct *C. arabica* accessions frequently correspond to genetically differentiated entities (Pruvot-Woehl et al., 2020; Montagnon and Serito, 2024; Montagnon et al., 2025b), we expected that the four morphotypes would differ in genetic identity. We further predicted that architectural traits, given their documented genotype-dependence in *C. arabica* (Rakočević, 2025), would provide greater discriminant capacity than leaf functional traits among the morphotypes studied.

## 2 Materials and methods

### 2.1 Study site and plant material

This study was conducted at VINKA Coffee Farm & Lab, a specialty coffee farm located in Loja province, southern Andes of Ecuador (4°19′19″S, 79°12′16″W; 1,660 m a.s.l.), within the transition zone between dry inter-Andean scrub and cloud forest, southwest of Podocarpus National Park. The site experiences a mean annual temperature of approximately 19 °C (range 14–25 °C) with a pronounced dry season from June to November, based on records from a HOBO meteorological station installed at the farm. The plantation covers 1.75 ha of coffee with uniform spacing (1.5 m between plants, 2.5 m between rows) under a mixed agroforestry system. Shade trees, primarily *Vachellia macracantha* (Humb. & Bonpl. ex Willd.) Seigler & Ebinger, a native legume of the inter-Andean dry scrub of southern Ecuador with documented benefits for soil nitrogen fixation, organic matter supply, and soil carbon and nitrogen stock enhancement in agroforestry systems (Abad et al., 2023), were established simultaneously with the coffee crop at a spacing of 10 m and had reached partial canopy coverage at the time of data collection, resulting in heterogeneous light conditions across the plantation. The cultivation system combines conventional and organic management practices under a syntropic agriculture framework, with mixed fertilization (organic and chemical) applied quarterly. Supplemental irrigation is provided during the dry season. Plants had not undergone pruning prior to data collection. The seeds labeled as Sidra were purchased from a private farm in Ecuador that maintains mother plants of this cultivar. The farm was identified through recommendations from other specialty coffee producers. The seeds labeled as Gesha were acquired from a coffee grower who imported the material from Panama. The grower was identified through social media networks. Neither source had formal commercial registration, official nursery certification, or phytosanitary documentation, and neither provided genetic certification or any traceability record for the planting material at the time of purchase. Purchase prices were USD 100/kg for Sidra and USD 500/kg for Gesha. Seeds were germinated and transplanted to field conditions following standard nursery protocols, and plants were grown for three years prior to data collection.

**Figure 1:**
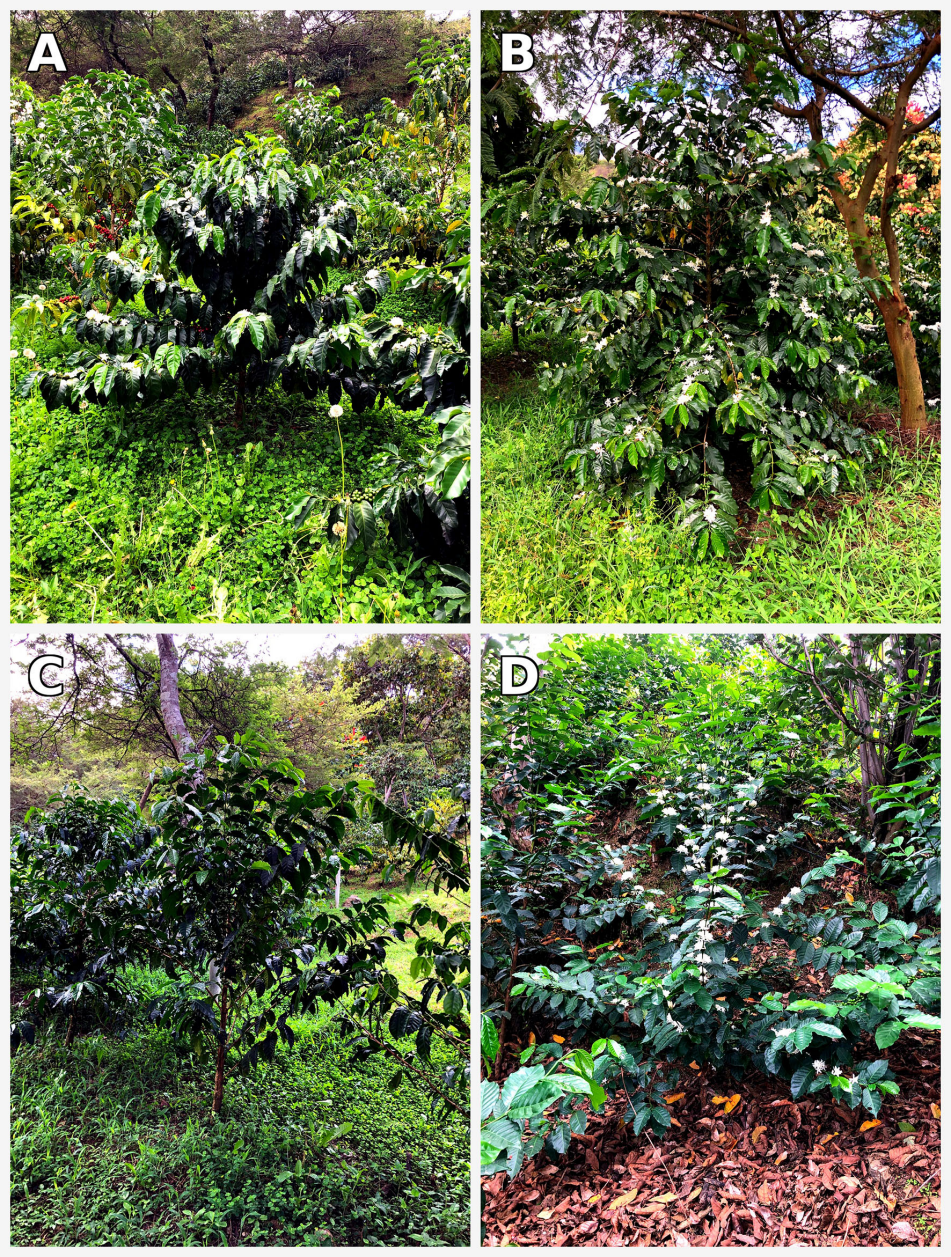
Morphological diversity of *Coffea arabica* cultivars at VINKA Coffee Farm, southern Ecuador. Representative field photographs of the four morphotypes under study. **(A)** Vinka-01: compact and densely branched shrub with abundant flowering (allelic profile compatible with Batian, Intergroup). **(B)** Vinka-02: tall plant architecture with semi-horizontal branching and profuse white flowers (confirmed as Sidra, Core Ethiopia). **(C)** Vinka-03: tall and slender growth form with an erect main axis and sparse lateral branching (confirmed as Sidra, Core Ethiopia). D) Vinka-04: tall, spreading canopy with large, dark-green leaves and white blossoms (pure Ethiopian landrace, Core Ethiopia; provisionally designated ‘Evangelina’). All photographs were taken during the 2025 flowering season.

After three years of field growth, three morphologically distinct groups were observed within the Sidra lot (approximately 4,000 plants total), hereafter designated Vinka-01, Vinka-02, and Vinka-03 (**Figures 1A–C**). These groups emerged gradually during plant development and were delimited based on the consistent and clear expression of contrasting phenotypic traits across multiple individuals, specifically plant stature and overall architecture, branching pattern and density, internode length, and fruit morphology. Within each group, individual plants were phenotypically homogeneous and clearly distinguishable from plants belonging to the other groups, with no intermediate phenotypes observed between groups. The delimitation of morphotypes was validated by an external coffee specialist (R. Coronel, pers. comm.) prior to formal sampling. Morphotypes Vinka-01, Vinka-02, and Vinka-03 were spatially intermixed throughout the plantation. Plants acquired as Gesha (approximately 250 plants; Vinka-04; **Figure 1D**) appeared phenotypically homogeneous across the entire lot and occupied a distinct sector within the farm. Together, these four groups constituted the four morphotypes evaluated in this study. The motivations for genetic verification were twofold: to determine whether intra-lot morphological divergence in Sidra represented true genetic differentiation, and to verify Gesha identity given the well-documented susceptibility of this variety to mislabeling (Pruvot-Woehl et al., 2020).

### 2.2 SSR-based genetic analysis

For molecular characterization, leaf tissue was collected from one representative individual per morphotype. Representative individuals were selected based on centrality of morphological expression within each group (i.e., individuals exhibiting the most typical traits of the group, avoiding atypical outliers). We acknowledge that this selection criterion introduces a potential circularity, since morphological typicality was used to both delimit morphotypes and identify individuals for genotyping. However, this approach maximizes the probability that the genotyped individual reflects the group’s predominant genetic identity. Ten young leaves per individual were collected from the middle third of the canopy using gloves and sterilized tools (absolute ethanol) to prevent cross-contamination, stored in paper bags under cool conditions during transport, and shipped to the RD2 Vision laboratory (Montpellier, France) for analysis. Given the predominantly autogamous reproductive biology of *C. arabica*, which results in extremely low within-individual genetic heterozygosity and high genomic uniformity within pure-line accessions (Scalabrin et al., 2020; Salojärvi et al., 2024), single-individual sampling is an analytically defensible approach for SSR-based identity verification in this species, and has been adopted in analogous authentication studies (Pruvot-Woehl et al., 2020). This approach was adopted strictly for identity verification purposes and does not allow estimation of within-morphotype genetic diversity or characterization of the genetic composition of the entire lot. These limitations, together with the economic constraints of certified SSR fingerprinting at an external laboratory, are addressed below.

Genomic DNA was extracted from approximately 20 mg of dried leaf tissue and quantified fluorimetrically following the extraction and quantification protocol of the certifying laboratory (RD2 Vision; Montagnon et al. (2025a)). DNA fingerprinting was performed with eleven SSR markers (**Table 1**) selected from Pruvot-Woehl et al. (2020) and Montagnon et al. (2025a). Genetic identity assignment was performed by the certifying laboratory through direct allele-by-allele comparison of each morphotype’s SSR profile against a proprietary reference database of *C. arabica* accessions. A morphotype was assigned to a reference cultivar if its allelic profile showed no mismatches with the known allelic range across all eleven loci. For composite cultivars without a fixed reference profile (i.e. introgressed varieties such as Batian), assignment was based on compatibility with the documented allelic range of the cultivar as described by Pruvot-Woehl et al. (2020). Profiles that did not match any reference cultivar were classified by their genetic group affiliation within the established diversity structure of *C. arabica* (e.g. Core Ethiopia, Intergroup, Ethiopian Legacy, or Typica/Bourbon), as described in Montagnon et al (Montagnon et al., 2025b; Montagnon et al., 2025a).

**Table 1:** List of the simple sequence repeat (SSR) makers used for the DNA fingerprinting analysis to verify *Coffea arabica* cultivar identity.

SSR marker	Primer sequence forward (5′–3′)	Primer sequence reverse (5′–3′)	Size product (bp)
Sat-11	ACCCGAAAGAAAGAACCAA	CCACACAACTCTCCTCATTC	143–145
Sat-207	CAATCTCTTTCCGATGCTCT	GAAGCCGTTTCAAGCC	83–93
Sat-225	CATGCCATCATCAATTCCAT	TTACTGCTCATCATTCCGCA	283–317
Sat-235	TCGTTCTGTCATTAAATCGTCAA	GCAAATCATGAAAATAGTTGGTG	245–278
Sat-24	GGCTCGAGATATCTGTTTAG	TTTAATGGGCATAGGGTCC	167–181
Sat-244	GCATACTAAGGAATTATCTGACTGCT	GCATGTGCTTTTTGATGTCGT	278–316
Sat-254	ATGTTCTTCGCTTCGCTAAC	AAGTGTGGGAGTGTCTGCAT	221–237
Sat-29	GACCATTACATTTCACACAC	GCATTTTGTTGCACACTGTA	137–154
Sat-32	AACTCTCCATTCCCGCATTC	CTGGGTTTTCTGTGTTCTCG	119–125
Sat-41	AGTGTAACTTTAGTTCTTGC	ATTTAATGGGCATAGGGTC	138–160
Sat-47	TGATGGACAGGAGTTGATGG	TGCCAATCTACCTACCCCTT	135–169

### 2.3 Morphological and functional trait measurements

We used an observational study design with four morphotypes (Vinka-01 to Vinka-04) as groups. Morphotypes were identified by the authors based on field observation of contrasting phenotypic traits (plant architecture, branching pattern, and fruit form), and their delimitation was validated by an external coffee specialist (R. Coronel, pers. comm.). Morphotypes Vinka-01, Vinka-02, and Vinka-03 were spatially intermixed throughout the Sidra lot, with sampled individuals distributed across the full extent of the planting area. This spatial interdigitation reduces the risk of systematic environmental confounding among these three morphotypes, as individuals of different morphotypes shared broadly similar light, slope, and soil conditions within the same planting area. Vinka-04 occupied a distinct sector within the farm; while this spatial separation limits direct environmental comparability with the other morphotypes for morphological traits, it does not affect the genetic identity assignment, which is independent of field conditions. No formal environmental covariates were recorded at the individual plant level, and microenvironmental heterogeneity within the plantation was not statistically controlled. We acknowledge that this could be source of environmental pseudoreplication, especially for environment-sensitive functional traits such as SLA and LDMC. This limitation was minimized by design through the spatial interdigitation of morphotypes and the compact size of the plantation within a single elevational band (1,660 m a.s.l.). However, it cannot be fully excluded, and its implications are addressed below. In November 2025, plant architecture, leaf functional traits, and fruit characteristics were measured on 10 plants per morphotype (n = 40 total; see **Table 2**). To minimize confounding environmental effects, sampled individuals of each morphotype were distributed across different locations within the farm, encompassing variation in light exposure, slope, and soil conditions. Each plant constituted an independent replicate. The sample size for Vinka-01 was reduced to n = 9 due to plant mortality caused by *Fusarium* sp. In January 2026, branch architecture (internode length and branch insertion angle) was assessed on the same individuals. However, due to additional plant mortality, the sample size for Vinka-01 was further reduced to n = 4 for these traits. Only plants with complete measurements across both sampling periods were included in the Linear Discriminant Analysis (n = 34: Vinka-01 = 4; Vinka-02, -03, -04 = 10 each). All measurements followed standardized protocols for plant functional traits (Abad et al., 2023; Pérez-Harguindeguy et al., 2013).

**Table 2:** Morphological trait means ± standard deviations for the morphotypes of *C. arabica* cultivars at VINKA Coffee Farm in southern Ecuador.

Trait	Vinka-01 (n = 9)	Vinka-02 (n = 10)	Vinka-03 (n = 10)	Vinka-04 (n = 10)
Plant volume (m³)	4.5 ± 2.7	7.7 ± 2.7	7.2 ± 2.7	6.1 ± 1.7
Fruit volume (mm³)	1166.2 ± 187.2	1071.9 ± 188.1	1136.4 ± 205.4	1217.6 ± 58.6
Fruit weight (g1)	21.0 ± 5.0	20.3 ± 4.0	21.6 ± 3.4	24.6 ± 0.9
Fresh leaf weight (g1)	27.1 ± 6.1	21.9 ± 2.6	19.9 ± 4.6	24.1 ± 2.4
LDMC (mg·g^-^1)	0.4 ± 0.0	0.4 ± 0.0	0.4 ± 0.0	0.4 ± 0.0
SLA (cm²·g^-^1)	9.7 ± 1.5	9.5 ± 0.9	10.4 ± 1.2	9.3 ± 0.9
Middle internode (cm)*	4.4 ± 1.2	4.9 ± 0.8	4.7 ± 0.6	6.5 ± 0.6
Lower internode (cm)*	3.9 ± 0.4	5.0 ± 0.6	5.0 ± 0.6	5.9 ± 0.5
Middle angle (°)*	45.8 ± 2.8	42.9 ± 6.9	50.7 ± 5.5	47.3 ± 5.3
Lower angle (°)*	52.9 ± 10.0	48.6 ± 9.2	53.1 ± 5.3	52.2 ± 6.7

LDMC, Leaf dry matter content; SLA, Specific leaf area. Asterisks (*) denote traits measured in only four individuals for morphotype Vinka-01, due to partial plant mortality caused by *Fusarium* infection.

#### 2.3.1 Plant architecture

Plant height (cm) and maximum and perpendicular canopy diameters (d_1_ and d_2_, cm) were measured on each individual. Plant volume (m³) was approximated as an elliptic cylinder: V = π × (d_1_/2) × (d_2_/2) × h, where h is plant height. Although this constitutes a geometric simplification of the irregular branching architecture of *C. arabica*, the elliptic cylinder provides a reproducible and field-applicable proxy for canopy volume that has been used in comparable *C. arabica* architectural studies (Matsunaga et al., 2016; Rakočević, 2025). The approach is appropriate for comparative purposes among morphotypes grown under similar conditions, as the objective was not to estimate absolute canopy volume but to derive a standardized size index sensitive to between-morphotype differences in plant stature and lateral spread.

#### 2.3.2 Leaf functional traits

Ten fully expanded, sun-exposed leaves were randomly collected per plant (100 leaves per morphotype) during the peak phenological stage of each plant, to minimize ontogenetic variation. Fresh leaf weight (g) was recorded immediately after collection to avoid water loss. Leaf area (LA, cm²) was determined from digital images using ImageJ software (Abramoff et al., 2004). Leaves were oven-dried at 80 °C for 48 h, and dry mass was recorded. Specific leaf area (SLA, cm²·g^-1^) was calculated as leaf area divided by leaf dry mass. Leaf dry matter content (LDMC, mg·g^-1^) was calculated as dry mass divided by fresh mass.

#### 2.3.3 Branch architecture

Branch architecture was assessed using a four-cardinal-direction protocol. For each plant, one primary plagiotropic branch was selected at each cardinal direction (N, S, E, W) at two canopy strata: middle (40–60% of plant height) and lower (10–30% of plant height). The apical stratum was excluded because branches in active elongation zones exhibit higher phenological variability and are less representative of the genotype-dependent architectural pattern (Matsunaga et al., 2016; Rakočević, 2025). At each selected branch, the two most basal internodes were measured. Basal internodes were specifically selected because they represent the most developmentally stable positions on plagiotropic branches in *C. arabica*, exhibiting lower phenological variability than distal internodes and reflecting the genotype-dependent elongation pattern of the branch more reliably (Rakočević, 2025). Internode length (cm) was recorded with a ruler, and branch insertion angle (°) relative to the main stem was measured with a digital protractor. Mean values per stratum were calculated across the four cardinal branches for each plant.

#### 2.3.4 Fruit characteristics

Ripe fruits were collected from the same 10 individuals used for leaf and architectural measurements (n = 10 fruits per plant, 100 fruits per morphotype), during peak ripeness. Fruit length, width, and thickness (mm) were measured with digital calipers, and fresh weight was recorded with a precision balance (± 0.001 g). Fruit volume (mm³) was approximated as an ellipsoid: V = (4/3) × π × (l/2) × (w/2) × (t/2), where l, w, and t are length, width, and thickness, respectively.

### 2.4 Statistical analysis

All statistical analyses were conducted in R language and environment for statistical computing (R Development Core Team, 2025). Morphological traits were summarized as mean ± standard deviation per morphotype. Normality of residuals was assessed using the Shapiro-Wilk test. All traits met the normality assumption and were analyzed using linear models (LMs) fitted with Type II sums of squares to account for the unbalanced design (Vinka-01 n = 9; Vinka-02–04 n = 10). Pairwise differences among morphotypes were assessed using Tukey-adjusted *post-hoc* contrasts (α = 0.05) implemented with the emmeans package (Russell et al., 2023). Due to the small sample size of Vinka-01 (n = 4) for architectural traits, its contribution to the LDA solution should be interpreted cautiously. A sample size of four may affect the stability of the discriminant functions and produce LOOCV-based classification accuracy estimates with wide uncertainty bounds. Therefore, results for Vinka-01 are reported descriptively and should not be directly compared with those of the remaining morphotypes.

To assess the multivariate discriminant capacity of morphological traits, Linear Discriminant Analysis (LDA) was performed using the MASS package (Venables and Ripley, 2002). The set of predictors was determined after evaluation of multivariate assumptions (see below), resulting in eight standardized traits: plant volume, fruit weight, fresh leaf weight, LDMC, SLA, middle internode length, lower internode length, and middle branch insertion angle. LDA identifies linear combinations of traits that maximize between-group variance relative to within-group variance. Classification accuracy was estimated using leave-one-out cross-validation (LOOCV), which provides unbiased accuracy estimates for small sample sizes. Discriminant loadings were used to identify traits contributing most to morphotype separation (Arias-Suárez et al., 2025).

The three assumptions underlying LDA were evaluated prior to analysis. First, collinearity among predictors was assessed using Pearson correlation coefficients and variance inflation factors (VIF) using the car package (Fox and Weisberg, 2011). One pair of variables exceeded the |r| = 0.80 threshold (fruit volume and fruit weight: r = 0.91, VIF ≈ 13), reflecting the fact that fruit volume is derived from fruit dimensions. Retaining both variables produced unstable discriminant coefficients, indicative of multicollinearity. Fruit volume was therefore excluded, while fruit weight was retained as a direct measurement. The final model included eight predictors, with all pairwise correlations ≤ |0.68| (maximum: SLA–LDMC) and all VIF values < 3 (maximum = 2.7), indicating no problematic collinearity. Second, homogeneity of within-group covariance matrices could not be assessed using Box’s M test because the smallest morphotype (Vinka-01; n = 4) had fewer observations than predictors, resulting in a singular covariance matrix. LDA was therefore fitted using a pooled within-group covariance matrix. Third, multivariate normality of the pooled within-group residuals was evaluated using Mardia’s and Henze–Zirkler tests implemented in the MVN package (Korkmaz et al., 2014). Neither test detected significant departures from multivariate normality (Mardia skewness p = 0.63; Mardia kurtosis p = 0.59; Henze–Zirkler HZ = 0.71, p = 1.00), consistent with the univariate Shapiro–Wilk results. Although these tests have limited power at n = 34, they provided no evidence of assumption violations.

## 3 Results

### 3.1 Genetic identity of the four morphotypes

SSR fingerprinting with eleven microsatellite markers resolved the genetic identity of all four morphotypes and placed each within established *C. arabica* diversity groups (**Supplementary Figure 1**; **Supplementary Table 1**). Vinka-02 and Vinka-03 matched the Sidra cultivar within the Core Ethiopia group [one of the two main genetic clusters of pure *C. arabica* landraces originating from Ethiopia; see the Arabica coffee Cultivars Wheel (Montagnon et al., 2025b);] (Montagnon and RD2 Vision, 2023; Montagnon et al., 2025b), confirming that the two morphologically distinct plants share a common genetic identity. Vinka-04 was identified as a pure Ethiopian landrace within the Core Ethiopia group. Its allelic profile differed from the Gesha reference accession T.02722 (CATIE) at 2 of the 11 SSR loci evaluated, specifically at Sat-225 (Vinka-04: 265–296; T.02722: 269–296) and Sat-24 (Vinka-04: 173–173; T.02722: 153–173). No matching profile was found across the vast RD2 Vision Lab reference database of *C. arabica* accessions. The complete allelic profiles for all morphotypes are provided in **Table 3**. Vinka-01 clustered unambiguously in the Intergroup zone [a genetic zone encompassing *C. arabica* varieties carrying introgressed *C. canephora* genes through the Timor Hybrid, distinct from pure *C. arabica* groups (Montagnon et al., 2025b);], a placement that is independent of any specific cultivar assignment and indicates this morphotype belongs to a genetically distinct background from the Core Ethiopia group. Within this zone, its allelic profile is compatible with the Batian cultivar selected in Kenya. Batian is a composite cultivar assembled from multiple lines and therefore does not have a single fixed DNA reference profile. The Intergroup placement of Vinka-01 is entirely distinct from the pure *C. arabica* background of the Core Ethiopia group. The certifying laboratory accordingly reports this assignment as compatibility rather than exact match.

**Table 3:** SSR allelic profiles of the four *C. arabica* morphotypes from VINKA Coffee Farm (southern Ecuador) across the 11 microsatellite markers used for genetic identity verification.

SSR marker	Vinka-01	Vinka-02	Vinka-03	Vinka-04
	(Batian-compatible)	(Sidra)	(Sidra)	(Ethiopian landrace)
Sat-11	145/145	145/145	145/145	145/145
Sat-207	83/89	83/93	83/93	83/93
Sat-225	265/296	269/296	269/296	**265/296**
Sat-235	227/227	265/265	265/265	257/257
Sat-24	163/163	167/167	167/167	**173/173**
Sat-244	278/302/306†	278/306	278/306	278/302
Sat-254	218/218	218/218	218/218	218/218
Sat-29	119/133	119/135	119/135	119/135
Sat-32	105/111	105/121	105/121	105/137
Sat-41	150/150	153/153	153/153	159/159
Sat-47	117/150	117/137	117/**147**	117/150

Alleles are reported as fragment sizes in base pairs. Bold values in the Vinka-04 column indicate the two loci (Sat-225 and Sat-24) at which this morphotype differs from Gesha reference accession T.02722 (CATIE), as confirmed by direct communication with the certifying laboratory (Dr. C. Montagnon, pers. comm.). †Three alleles were detected at locus Sat-244 for Vinka-01, an unusual pattern in the predominantly autogamous *C. arabica* that may reflect residual heterozygosity at this locus in the introgressed genetic background of this morphotype. Bold value in the Vinka-03 column at Sat-47 indicates a one-locus difference from Vinka-02; both morphotypes were independently confirmed as compatible with Sidra by the certifying laboratory. Genetic group assignments: Vinka-01, Intergroup (allelic profile compatible with Batian); Vinka-02 and Vinka-03, Core Ethiopia (Sidra); Vinka-04, Core Ethiopia (pure Ethiopian landrace, provisionally designated ‘Evangelina’).

### 3.2 Morphological trait variation among morphotypes

Morphological traits (**Table 2**) differed significantly among morphotypes for six of the ten measured variables (**Supplementary Table 2**). Internode length was the most strongly differentiated trait, showing highly significant differences at both canopy strata (p < 0.001 for middle and lower internodes; **Supplementary Table 2**), and ordering across strata. Vinka-04 exhibited the longest internodes at both the middle (6.5 ± 0.4 cm) and lower (5.9 ± 0.5 cm) strata, significantly exceeding all other morphotypes in all pairwise comparisons (**Supplementary Table 3**), while Vinka-01 showed the shortest internodes at both strata (middle: 4.4 ± 1.2 cm; lower: 3.9 ± 0.4 cm). Notably, Vinka-02 and Vinka-03, the two morphotypes sharing a confirmed Sidra genetic identity, did not differ from each other in internode length at either stratum, while both differed from Vinka-04 and partially from Vinka-01 (**Supplementary Table 3**). This pattern of internode length variation was thus concordant with the SSR-based genetic groupings across both canopy strata. Among the remaining traits, fresh leaf weight, LDMC, plant volume, and middle branch insertion angle also differed significantly among morphotypes (p < 0.05 for all; **Supplementary Table 2**). Vinka-01 showed the highest fresh leaf weight (27.1 ± 6.1 g) and smallest plant volume (4.5 ± 2.7 m³), suggesting a more compact architecture with denser leaf tissue, while Vinka-02 showed the largest plant volume (7.7 ± 2.7 m³) and higher LDMC (0.393 ± 0.025 mg g^-^¹) relative to Vinka-03 (0.356 ± 0.016 mg g^-^¹). SLA, fruit volume, fruit weight, and lower branch insertion angle showed no significant differences among morphotypes. Pairwise contrasts for all traits are provided in **Supplementary Table 3** and distributions are shown in **Supplementary Figure 2**.

### 3.3 Multivariate morphological discrimination

**Figure 2:**
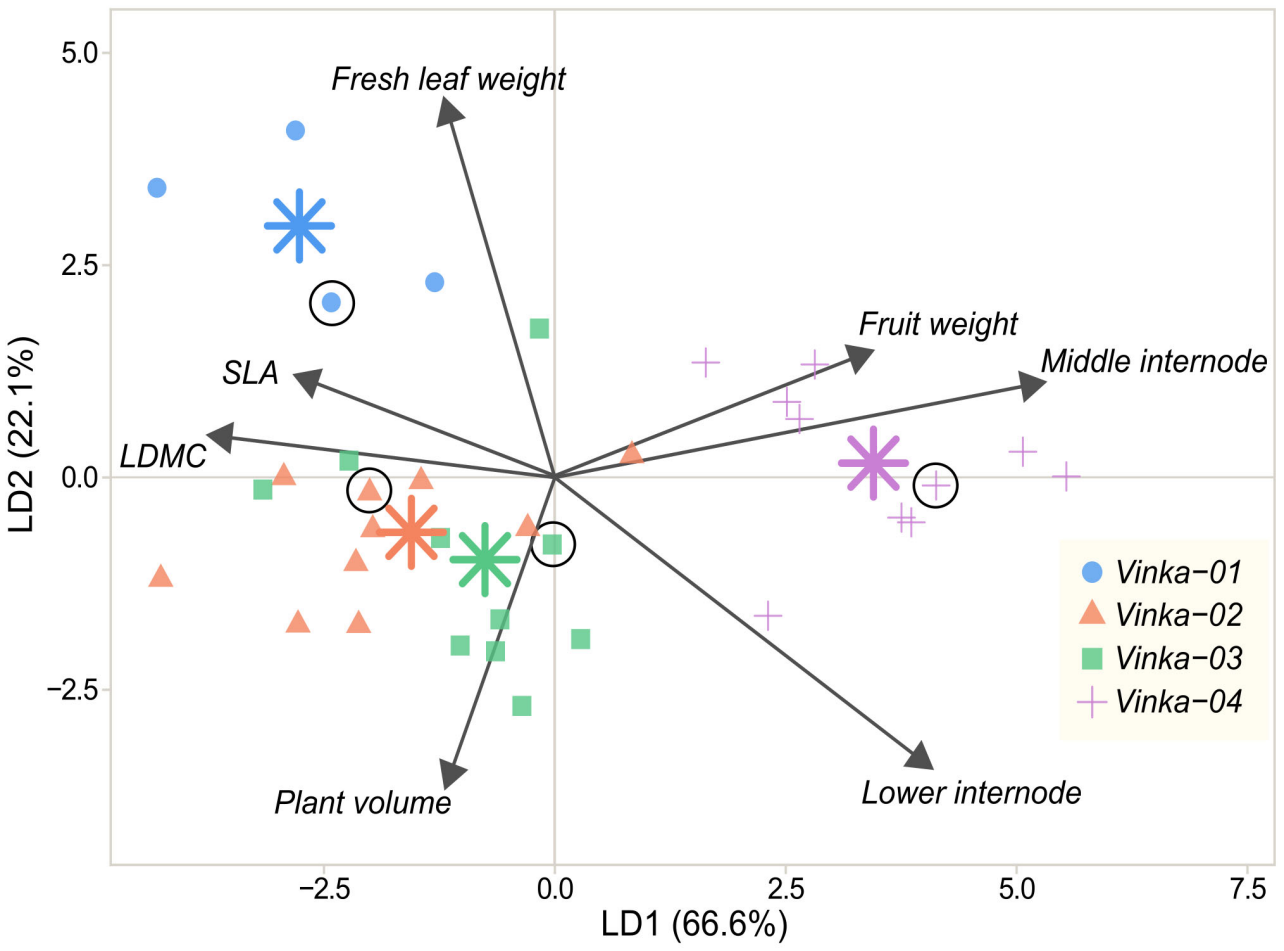
Linear discriminant analysis biplot showing morphological separation among *C*. *arabica* morphotypes from VINKA Coffee Farm, southern Ecuador. Points represent individual plants; asterisks indicate morphotype centroids; circled points identify the representative individuals selected for SSR-based genetic analysis (see **Supplementary Figure 1** for genetic results). Vectors show discriminant loadings of morphological traits. LD1 (66.6%) separates morphotypes primarily by internode length, driving the rightward separation of Vinka-04; LD2 (22.1%) is dominated by fresh leaf weight, separating Vinka-01 (upper quadrant) from Vinka-02 and Vinka-03 (lower quadrant). Vinka-04 achieves complete separation from all other morphotypes, while Vinka-02 and Vinka-03 overlap in discriminant space, consistent with their shared genetic identity as Sidra cultivar. Genetic identities by morphotype: Vinka-01, allelic profile compatible with Batian (Intergroup); Vinka-02 and Vinka-03, confirmed as Sidra (Core Ethiopia); Vinka-04, pure Ethiopian landrace provisionally designated ‘Evangelina’ (Core Ethiopia).

LDA using eight standardized traits correctly classified 82.4% of plants (28/34) under leave-one-out cross-validation (**Supplementary Table 4**). Classification accuracy was perfect for Vinka-04 (100%; 10/10), moderate for Vinka-03 (80%; 8/10) and lowest for Vinka-02 (70%; 7/10). For Vinka-01, three of four plants were correctly classified (75%); however, given the very small sample size for this morphotype, this estimate carries wide uncertainty and should not be compared directly with the accuracy values of the remaining groups. The first two discriminant functions collectively explained 89.3% of total between-group variance (LD1: 66.6%; LD2: 22.1%; **Figure 2**). Internode length (middle and lower) generated the largest loadings along LD1, driving the rightward separation of Vinka-04 from the remaining morphotypes. Along LD2, fresh leaf weight loaded positively, separating Vinka-01 (upper quadrant) from Vinka-02 and -03 (lower quadrant), while plant volume loaded negatively. SLA and LDMC generated moderate negative loadings along LD1, contributing to the leftward placement of Vinka-01. Vinka-02 and Vinka-03 occupied largely overlapping positions in discriminant space, with misclassifications occurring exclusively between these two groups (four plants total). This overlap is consistent with their shared genetic identity as Sidra, which lacks a fixed reference fingerprint and encompasses intraspecific morphological variation. Vinka-04, uniquely characterized by long internodes and heavier fruits, was the only morphotype fully separated from all others.

## 4 Discussion

### 4.1 Cultivar misidentification: morphological and genetic evidence

This study provides the first documented evidence of varietal misidentification in commercially acquired specialty *C*. *arabica* planting material at the farm scale in Ecuador. By combining SSR-based genetic fingerprinting with quantitative morphological characterization, we show that two of the four morphotypes analyzed were inconsistent with their declared commercial identity. One morphotype sold as Sidra showed an allelic profile compatible with Batian, a composite variety of Kenyan origin from a genetically distant breeding background; while single-individual sampling precludes a definitive cultivar-level assignment, the placement of this morphotype in the Intergroup zone is unambiguous and confirms the presence of introgressed *C. canephora* genes absent from the Core Ethiopia group. The seeds acquired as Gesha corresponded to a pure Ethiopian landrace that, while genetically affiliated with the Core Ethiopia group, was not identical to the Panamanian Geisha reference accession T.02722. Only two morphotypes matched the declared commercial name.

Morphological assessment captured part of this genetic diversity, with branch internode length emerging as the trait with the greatest discriminant capacity, consistent with our hypothesis. Together, these findings illustrate that varietal non-conformity in the specialty coffee seed sector is not limited to within-variety genetic drift but can extend to the inadvertent introduction of genetically unrelated plant material, a distinction with direct implications for producers, buyers, and regulators alike.

The most striking result of this study is the placement of the Vinka-01 morphotype in the Intergroup zone, with an allelic profile compatible with Batian, a Kenyan cultivar deriving from complex crosses involving introgression with the Timor Hybrid. Regardless of the specific cultivar-level assignment, this morphotype is unambiguously genetically remote from the Core Ethiopia group to which true Sidra cultivar belongs (Pruvot-Woehl et al., 2020; Montagnon et al., 2025b). It should be noted, however, that the Intergroup zone encompasses multiple cultivars with introgressed *C. canephora* backgrounds, including Ruiru 11, various Catimors, and Sarchimors, some of which share allelic profiles that are difficult to distinguish at the resolution of 11 SSR markers. The assignment to Batian specifically reflects the assessment of the certifying laboratory based on the combination of alleles observed and its compatibility with the known allelic range of this composite cultivar, but we cannot exclude that the profile could be consistent with another Intergroup variety not represented in the reference database. What remains unambiguous and is sufficient to establish the core finding of this study, is that Vinka-01 carries introgressed *C. canephora* genes and belongs to a genetic background entirely incompatible with the declared Sidra identity. The presence of such genetic pattern in a lot commercially sold as Ecuadorian Sidra represents a qualitatively different form of non-conformity compared to, for example, the admixture of related Ethiopian accessions under the same commercial name. Although cup profile and productive performance were not directly evaluated in this study, prior work has documented that sensory and agronomic attributes in *C. arabica* are partially dependent on genetic identity (Louzada MaChado et al., 2022; Arias-Suárez et al., 2025). Based on this, it can be expected that within-group variation may preserve some aspects of the expected sensory or agronomic performance. However, the substitution of material from a different genetic background introduces uncertainty about these attributes that cannot be resolved without a sensory and yield evaluation of the specific material involved. The plant architecture differences between Vinka-01 and the confirmed Sidra morphotypes documented in this study, however, are directly evaluated and constitute objective evidence that this material does not correspond to the architecture buyers and producers associate with Sidra. This outcome is consistent with the broader trend documented by Montagnon and Serito (2024), who found that about one-third of the coffee plants submitted by growers from 24 countries did not match the declared variety, and that cultivars traded under commercial names without fixed genetic reference profiles were among the most frequently misidentified. This is precisely the situation with the Vinka-01 morphotype, whose Intergroup placement is inconsistent with the declared Sidra identity regardless of its specific cultivar-level assignment, and Vinka-04, whose Core Ethiopia profile does not correspond to the Panamanian Gesha lineage.

Morphologically, Vinka-01 was the most compact morphotype in this study, exhibiting the shortest internodes at both canopy strata and the highest fresh leaf weight. These traits were distinguishable from those of the other two Sidra morphotypes, particularly after three years of field growth. These findings suggest that attentive phenotypic monitoring by an informed producer or agronomist could identify this type of outlier early on, even without molecular tools. However, the subtle differences between Vinka-02 and Vinka-03 morphotypes, which are all phenotypically consistent with expectations for high-altitude Ethiopian-origin varieties, demonstrate that visual screening alone is insufficient to reveal the full extent of nonconformity. Together with the overlap of Vinka-02 and Vinka-03 in the LDA discriminant space, as discussed below, this observation reveals a partial decoupling between morphological and genetic differentiation within the Sidra cultivar itself. Two plants with the same SSR-based genetic identity produced detectable differences in fresh leaf weight, leaf dry matter content (LDMC), plant volume, and middle branch insertion angle. This decoupling confirms that the Sidra cultivar, as currently traded, is not morphologically uniform but rather encompasses measurable intraspecific variation. This is consistent with its absence of a fixed reference profile and its likely status as a selected Ethiopian landrace. From an applied perspective, this empirical feature of the cultivar must be incorporated into any future authentication protocol since cultivar-level identity within the Core Ethiopia group cannot be reliably resolved by morphology alone.

The placement of the Sidra Vinka-02 and Vinka-03 morphotypes within the Core Ethiopia genetic group is consistent with prior genetic-level analyses that place Sidra within this cluster (Montagnon and RD2 Vision, 2023), and adds two farm-level observations to that body of evidence. As noted in the Introduction section, a hybrid origin involving different *C. arabica* lineages would be expected to produce allelic profiles intermediate between any group and the Core Ethiopian cluster, or to place the variety within the introgressed genetic zone. Neither of these scenarios is observed here. While based on only two genotyped individuals from a single site, these observations agree with the interpretation, drawn from broader genetic studies, that Sidra cultivar represents a selected Ethiopian landrace rather than a variety derived from human-directed breeding programs. This interpretation should be confirmed through population-level sampling of commercially available Sidra material across multiple origins. Three broader implications follow from this interpretation. From an evolutionary perspective, placing Sidra within Core Ethiopia suggests that the intraspecific variation observed under this commercial name is the result of incidental selection on a subset of Ethiopian landrace diversity rather than directed breeding from defined parental lines. This aligns Sidra with other commercially available landrace-derived materials whose phenotypic heterogeneity reflects the genetic structure of their source population (Tesfaye et al., 2014; Montagnon et al., 2025b). From a breeding perspective, this status indicates that Sidra is a valuable reservoir of Core Ethiopian diversity for genetic improvement programs. However, it cannot be used as a defined parental line in controlled crosses without first characterizing the specific material’s genotype. From a commercial perspective, our results suggest that the certification and commercialization of Sidra should be reconsidered. Unlike Gesha, which can be authenticated using a single reference genotype (T.02722), Sidra appears to be a group of closely related Ethiopian-derived materials rather than a single uniform cultivar. Thus, authenticating Sidra may require a broader approach combining SSR markers confirming its Core Ethiopia origin and morphological descriptors that capture the variation observed among Sidra accessions.

The result for morphotype Vinka-04 illustrates a distinct but equally consequential form of identity discordance. The plant acquired and grown as Gesha was confirmed as a pure Ethiopian landrace (Core Ethiopia group), genetically proximate to but not identical with the Panamanian Geisha reference accession T.02722. This finding aligns closely with the pattern documented by Pruvot-Woehl et al. (2020), who reported that only 39% of samples globally submitted as Gesha matched its reference genotype, and that a substantial fraction corresponded instead to unrelated Ethiopian landraces circulating under the same commercial name. Because Gesha commands some of the highest prices in the international specialty coffee market (Batista Ledezma et al., 2025), a value rooted in the replicability of the sensory profile associated with the T.02722 lineage, this level of identity uncertainty poses a direct traceability risk (Pruvot-Woehl et al., 2020). The phenotypic homogeneity of the Vinka-04 morphotype within the farm lot is consistent with that of a pure landrace accession. However, this does not indicate a problem; an unverified Ethiopian landrace may produce a distinctive and commercially valuable cup. The issue is the inability to make verified claims about the genetic origin, and therefore about the expected sensory and agronomic profile, when the genetic identity of the material has not been confirmed through molecular analysis prior to planting. The instability of the commercial name ‘Gesha’ as a genetic guarantor, documented globally, is evidently replicated at the individual farm level in Ecuador.

Identifying Vinka-04 as a pure Ethiopian landrace that differs from the Gesha reference accession (T.02722) raises an additional point of interest regarding the origin and traceability of this material. It is unlikely that this profile corresponds to a mislabeled accession from an existing, unsampled collection. The absence of a matching genotype in the certifying laboratory’s extensive *C. arabica* reference database suggests that Vinka-04 is not closely associated with any currently documented accession. This suggests that the material represents a previously uncharacterized genetic lineage within specialty coffee varietals (pers. comm. Dr C. Montagnon). A more plausible interpretation, consistent with the commercial history of this seed lot, is that this accession represents Ethiopian genetic material that entered the Ecuadorian seed supply through informal channels under the commercial name ‘Gesha’ without corresponding to the Panamanian T.02722 lineage. Regardless of its precise origin, this material currently lacks a formal designation or traceability within existing certification frameworks, and its allelic profile has not been previously reported in the literature. Pending broader morphological and genetic characterization across multiple individuals and growing sites, we propose the provisional field name ‘*Evangelina*’ for this plant material solely for farm-level traceability purposes at VINKA Coffee Farm. This designation follows the specialty coffee sector’s established practice of documenting and tracking accessions before formal variety registration (World Coffee Research, 2023a) and should not be interpreted as a formal taxonomic or varietal designation. Formal registration would require characterization of a larger population, multi-environment evaluation and submission to an official variety protection body. We acknowledge the concern that a provisional name may circulate commercially before the necessary formal characterization is complete, which could generate confusion in the specialty coffee sector. To minimize this risk, we explicitly caution against using ‘*Evangelina*’ in any commercial, marketing, or seed trading context before formal varietal registration. We encourage any party with access to Ethiopian germplasm collections or broader SSR reference data to collaborate on the characterization necessary to establish or supersede this provisional designation.

The morphological results support our initial hypothesis that architectural traits have greater discriminant capacity than leaf functional traits among genetically distinct *C. arabica* morphotypes. Internode length dominated the first linear discriminant function, and Vinka-04 (the morphotype with the longest internodes at both canopy strata) was the only group that was perfectly classified using LOOCV (100%). The dominance of internode length on LD1 reflects its direct dependence on genetically controlled patterns of meristematic elongation in plagiotropic branches, a process less buffered by environmental conditions than leaf-level traits such as SLA or LDMC (Matsunaga et al., 2016; Rakočević, 2025). Internode elongation rate and final internode length are determined by the activity of intercalary meristems during branch ontogeny, and inter-cultivar variation in this trait reflects genotypic differences in hormonal control of cell elongation rather than plastic responses to current environmental conditions (Rakočević, 2025). This explains why architectural traits provide a more stable discriminant signal than leaf functional traits under uncontrolled field conditions. This suggests that the unusually long internodes characteristic of the Ethiopian landrace in this group is a reliable, field-accessible phenotypic indicator. Therefore, the complete discriminant separation of Vinka-04 along LD1 is functionally meaningful because it identifies the Ethiopian landrace morphotype by its characteristic elongated, plagiotropic architecture. This phenotype is consistent with the architectural variation documented within the Core Ethiopia group (Montagnon et al., 2025b; Rakočević, 2025). Landraces such as Gesha and related accessions are characterized by tall, open canopies with elongated internodes (World Coffee Research, 2023a). This contrasts with the more compact architectures of Typica/Bourbon-derived varieties or introgressed cultivars, such as Batian (Montagnon et al., 2025b; Rakočević, 2025). This contrast aligns with a domestication-related architectural gradient in *C. arabica*. Ethiopian landraces retain the open, vigorous canopy architecture characteristic of their ancestral forest-edge habitat (Tesfaye et al., 2014; Degefa et al., 2021). In this habitat, elongated internodes and tall plagiotropic branches optimize light capture in heterogeneous canopies. In contrast, modern bred cultivars have been selected for compact stature, which is suitable for higher planting densities and mechanized management (Montagnon et al., 2025b; Rakočević, 2025). Thus, internode length captures a genotypically stable trait and a domestication signal that distinguishes ancestral landrace architectures from human-shaped commercial varietal types. This architectural signature, although insufficient on its own to discriminate among landraces within the Core Ethiopia group, provides a robust first-tier indicator distinguishing this genetic background from the compact architectures associated with introgressed varieties. The consistency of this separation across both canopy strata, combined with the perfect classification accuracy achieved for Vinka-04 under LOOCV, suggests that internode length in mature plagiotropic branches is a phenotypically stable trait for this morphotype under the field conditions evaluated. From an applied perspective, this finding has direct implications for the field-based authentication of Gesha and related Ethiopian landrace varieties at the producer level. The measurement of internode length in the middle and lower canopy strata using the standardized protocol described here offers producers and agronomists an accessible, low-cost screening tool to identify Ethiopian landrace varieties for further molecular analysis. While such phenotypic screening cannot replace SSR-based authentication for commercially sensitive transactions, it provides an actionable first-tier filter in contexts where molecular fingerprinting is economically or logistically inaccessible. This finding is consistent with the established role of branch architecture as a genotype-dependent trait that reflects the genetics governing meristematic elongation in *C. arabica* (Rakočević, 2025). It is also consistent with the broader observation that architectural descriptors can distinguish between cultivars when leaf-level traits are insufficient (Sousa et al., 2017).

The overall LDA accuracy of 82.4% shows that including architectural traits in the multivariate model adds genuine discriminant value to quantitative morphological assessment. However, the significant overlap of the Vinka-02 and Vinka-03 morphotypes in discriminant space (both confirmed as Sidra) illustrates a fundamental limitation of morphology-based differentiation. This is particularly true for varieties lacking a fixed genetic reference profile that encompasses broad intraspecific variation (Montagnon et al., 2025b). The combination of higher fresh leaf weight and more compact plant volume observed in Vinka-01 relative to the confirmed Sidra morphotypes is consistent with the architectural differences expected between introgressed varieties and pure Ethiopian landraces, as Catimor and Sarchimor derivatives, which share genetic background with Batian, are generally characterized by more compact canopy architectures than Ethiopian landrace cultivars (Montagnon et al., 2025b). Leaf functional traits, specifically LDMC and SLA, which are key components of the leaf economics spectrum (Wright et al., 2004), exhibited statistically significant differences in univariate analyses but produced lower discriminant loadings in the multivariate analysis. The higher LDMC of Vinka-02 relative to Vinka-03, both confirmed as Sidra, likely reflects intraspecific variation in leaf water content rather than a genotypically meaningful difference, which is consistent with the documented sensitivity of these traits to local environmental factors such as light availability, management intensity, and phenological stage (Martin et al., 2017; García et al., 2025). This sensitivity may partially mask genotypic signals under non-controlled field conditions. In combination, these results suggest that morphological characterization is the most informative first-tier screening tool, especially when architectural traits are prioritized. However, molecular fingerprinting is still necessary to resolve ambiguous cases and confirm identity in commercially sensitive contexts (Nakagawa et al., 2019; Pruvot-Woehl et al., 2020; Bolívar-González et al., 2022; Andini et al., 2025).

Beyond documenting misidentification, this study offers preliminary morphological data with potential long-term value for Sidra authentication efforts. Unlike Gesha, which has a verifiable reference accession (T.02722) used for genetic authentication worldwide (Pruvot-Woehl et al., 2020), there is currently no equivalent morphological or genetic standard for Sidra cultivar. This complicates both field identification and formal variety protection under UPOV guidelines (IPGRI, 1996; UPOV, 2007). The quantitative morphological profiles reported here for two genetically confirmed Sidra plants provide an initial, exploratory characterization for this cultivar under field conditions at a single site. Given that these data derived from a one farm, two genotyped individuals, and a single growing environment, they should not be interpreted as a validated reference framework; their value lies in demonstrating the feasibility of a standardized measurement approach and in providing a preliminary baseline that could be replicated across multiple sites and a larger number of genotyped individuals. We suggest that the development of a formal descriptor list for Sidra, based on genetically verified material from diverse growing environments and following the UPOV framework, should be a priority for the specialty coffee sector.

### 4.2 Seed traceability and producer vulnerability

The economic stakes of the misidentification documented here are concrete and quantifiable. Seeds were purchased at USD 100 kg (Sidra) and USD 500 kg (Gesha) from recognized commercial suppliers in Ecuador, without any form of genetic certification or phytosanitary documentation, and the resulting non-conformity went undetected for at least three years of field growth before morphological divergence prompted formal verification (World Coffee Research, 2024). For a specialty coffee producer, the consequences of non-conformity extend well beyond the initial purchase price. Given the multi-year production cycle of *C. arabica*, which typically takes three to four years to reach significant harvests levels, investments in planting, canopy management, and variety-specific marketing accumulate under the assumption that the acquired material will express the expected genetic identity. When this does not occur, the economic and reputational burden falls disproportionately on the producer. Currently, there is no legal or technical mechanism within Ecuador’s specialty coffee seed trade through which producers can seek compensation for such losses. Unlike in Costa Rica, there is no government agency or germplasm bank that provides genetically certified seeds (Gatica-Arias et al., 2025). Furthermore, if the cup profile delivered at harvest differs from the contracted variety, questions of producer transparency and commercial credibility arise, even when the grower is the primary victim of an unregulated supply chain (Pruvot-Woehl et al., 2020). The VINKA farm case thus exemplifies a broader systemic problem, paying premium prices for certified-identity varieties in the absence of a system that can guarantee that identity at the point of sale.

### 4.3 Limitations and future directions

Several limitations of this study should be acknowledged when interpreting these findings. First, the genetic analysis was based on a single representative individual per morphotype, which is costly for coffee producers. While the predominantly autogamous reproductive biology of *C. arabica* limits intra-plant genetic heterogeneity and makes single-individual identity verification analytically defensible (Anthony et al., 2002; Scalabrin et al., 2020; Salojärvi et al., 2024), it does not allow for the estimation of genetic diversity within-morphotype. Increasing the number of genotyped individuals per morphotype or cultivar in future studies would strengthen conclusions about the homogeneity of each group (Andini et al., 2025). Additionally, this study represents a single farm case in southern Ecuador, and its findings should not be used to estimate the national or regional prevalence of non-conformity without broader, systematic sampling of the Ecuadorian specialty coffee seed sector. The morphological measurements were conducted at two sampling dates approximately two months apart. This introduces the possibility that seasonal growth affected the values of architectural traits relative to leaf and fruit traits (Rakocevic and Matsunaga, 2018; Unigarro et al., 2025). While the plants were at the same broad phenological stage during the flowering season, differential growth rates among genetically distinct morphotypes could have contributed to some of the observed variation. Finally, the reduced sample size for Vinka-01 morphotype in the architectural trait measurements (n = 4) limits the statistical precision of classification estimates for this morphotype.

Despite these limitations, this study provides a replicable methodological framework for identifying cultivar misidentification in specialty coffee at the farm scale. This framework combines field-accessible morphological characterization (mainly architectural traits) with SSR genotyping against a validated reference database. For Ecuador specifically, where the specialty coffee sector is expanding at high altitudes in Loja Province and neighboring regions, the documentation of a Kenyan composite variety and an unverified Ethiopian landrace within commercially sold Sidra and Gesha lots highlights the urgent need for accessible seed certification mechanisms. Investment in such infrastructure, such as the CheckCAFE program (World Coffee Research, 2023b) in few countries of Latin America, would reduce producer vulnerability, strengthen buyer trust, and provide the genetic traceability that premium specialty markets increasingly demand as part of their quality assurance protocols. As the commercial value of named C. arabica varieties continues to rise, and as producers and roasters in countries such as Ecuador invest in building reputations around specific genetic identities, the discrepancy between what farmers purchase and ultimately plant represents more than just individual economic risk. It signifies a systemic failure within the seed sector. Science-based verification tools are now more accessible than ever through partnerships with specialized laboratories. These tools are well positioned to close the gap in the specialty coffee seed sector, if there is an institutional will to formalize it that matches the scientific capacity to verify it.

## 5 Conclusions

This study shows that misidentification of specialty *C. arabica* planting material can extend beyond genetic drift within a variety to include material that is genetically unrelated. Only two of the four morphotypes analyzed matched their declared commercial identity: one morphotype sold as Sidra was identified as compatible with Batian, a Kenyan variety with introgressed *C. canephora* genes that are entirely unrelated to the Core Ethiopia group, and the morphotype acquired as Gesha corresponded to a pure Ethiopian landrace that is distinct from the Panamanian reference accession T.02722.

Morphological characterization proved to be an informative first-tier screening tool, with internode length being the most discriminating trait, but molecular fingerprinting remains necessary to confirm identity in commercially sensitive contexts. The quantitative morphological profiles reported here for two genetically verified Sidra plants represent a preliminary, single-site characterization that could serve as a starting point for the development of a standardized morphological reference for this cultivar, pending replication across multiple growing environments and a larger number of genotyped individuals. Such a resource is urgently needed to support traceability and certification in a specialty market where genetic identity and commercial value are inextricably linked. Investment in accessible seed verification mechanisms, such as the CheckCAFE program (World Coffee Research, 2023b) already operational in several Latin American countries, would provide producers in Ecuador and comparable contexts with the tools needed to verify varietal identity before planting, reducing the vulnerability documented in this study.

Taken together, the findings of this study lead to three broader conceptual conclusions. First, the case documented here highlights the structural fragility of informal seed supply systems in the specialty coffee sector. In the absence of regulated certification and traceability mechanisms, varietal non-conformity is not an isolated incident but a predictable consequence of how planting material is acquired and propagated. Second, the results reveal a disconnect between commercial and genetic identities. Cultivar names widely used in markets, contracts, and quality competitions are not always linked to verified genetic references, particularly in cultivars such as Sidra that lack a defined reference genotype. As a result, the risk of non-conformity is transferred to producers and downstream buyers, who often have no practical means of verifying the identity of the material they purchase or sell. Third, addressing this gap will require the formal incorporation of molecular authentication tools into specialty coffee certification frameworks. These tools should be accessible at the point of seed acquisition rather than restricted to specialized or elite producers. Without such measures, the premium value associated with specialty cultivars cannot be reliably linked to their genetic identity, leaving the certification systems that support the specialty coffee market vulnerable to the types of misidentifications documented in this study.

## Data Availability

The morphological and functional trait dataset generated and analyzed in this study is publicly available in the Figshare repository at 10.6084/m9.figshare.32841344.
