# Supplementary material for: What you plant may not be what you bought: morphological and genetic discordance in specialty Coffea arabica L. cultivars from Ecuador
Source: Front Plant Sci. 2026 Jul 14;17:1868034. doi: 10.3389/fpls.2026.1868034 (PMC13409818; doi:10.3389/fpls.2026.1868034)
Supplement: Supplementary file 1 [file Table1.docx]

Supplementary Material

# Supplementary Tables

**Table S1.** Summary of the SSR fingerprinting results confirming the genetic identity of *C. arabica* samples collected at VINKA coffee farm in southern Ecuador.

| **Sample ID** | **Confirmed cultivar** | **Genetic group** | **Observations** |
| --- | --- | --- | --- |
| Vinka-01 | Batian | Intergroup | The sample is genetically compatible with the Batian cultivar, a Kenyan variety carrying introgressed *C. canephora* genes through the Timor Hybrid, similar to Catimors and Sarchimors. As with other complex interspecific hybrids of this lineage, Batian lacks a single fixed DNA reference profile, which explains the observed combination of alleles. |
| Vinka-02 | Sidra | Core Ethiopia | The sample matches the Sidra cultivar. Genetically, Sidra is unrelated to Bourbon and belongs to the Ethiopian Landrace group. |
| Vinka-03 | Sidra | Core Ethiopia | The sample matches the Sidra cultivar. Genetically, Sidra is unrelated to Bourbon and belongs to the Ethiopian Landrace group. |
| Vinka-04 | Landraces | Core Ethiopia | The sample is a pure Ethiopian Landrace genetically close to, but distinct from, the Gesha reference accession (T.02722, CATIE), representing an as-yet-unnamed Ethiopian accession. Pending formal morphological and genetic characterization, the authors provisionally designate this accession 'Evangelina' for field traceability purposes at VINKA Coffee Farm. |

**Table S2.** Statistical tests comparing morphological traits among varieties. GLM = generalized linear model.

| **Trait** | **Test** | **p-value** |
| --- | --- | --- |
| Plant volume | GLM | 0.0366 |
| Fruit volume | GLM | 0.2962 |
| Fruit weight | GLM | 0.055 |
| Fresh leaf weight | GLM | 0.0044 |
| LDMC | GLM | 0.015 |
| SLA | GLM | 0.1895 |
| Middle internode | GLM | 0.000 |
| Lower internode | GLM | 0.000 |
| Middle insertion angle | GLM | 0.0384 |
| Lower insertion angle | GLM | 0.5487 |

**Table S3.** Tukey post-hoc pairwise comparisons for traits showing significant differences (p < 0.05 only).

| **Trait** | **Contrast** | **Estimate** | **SE** | **df** | **t-ratio** | **p-value** |
| --- | --- | --- | --- | --- | --- | --- |
| Plant volume | Vinka-01 - Vinka-02 | -3.292 | 1.145 | 35 | -2.88 | 0.0330 |
| Fruit weight | Vinka-02 - Vinka-04 | -4.298 | 1.586 | 34 | -2.71 | 0.0489 |
| Fresh leaf weight | Vinka-01 - Vinka-02 | 5.132 | 1.897 | 35 | 2.71 | 0.0490 |
| Fresh leaf weight | Vinka-01 - Vinka-03 | 7.140 | 1.897 | 35 | 3.76 | 0.0033 |
| LDMC | Vinka-02 - Vinka-03 | 0.037 | 0.011 | 35 | 3.33 | 0.0105 |
| Middle internode | Vinka-01 - Vinka-04 | -2.104 | 0.446 | 30 | -4.72 | 0.0003 |
| Middle internode | Vinka-02 - Vinka-04 | -1.536 | 0.337 | 30 | -4.56 | 0.0004 |
| Middle internode | Vinka-03 - Vinka-04 | -1.782 | 0.337 | 30 | -5.29 | 0.0001 |
| Lower internode | Vinka-01 - Vinka-02 | -1.076 | 0.322 | 30 | -3.34 | 0.0115 |
| Lower internode | Vinka-01 - Vinka-03 | -1.124 | 0.322 | 30 | -3.49 | 0.0079 |
| Lower internode | Vinka-01 - Vinka-04 | -2.027 | 0.322 | 30 | -6.29 | 0.0000 |
| Lower internode | Vinka-02 - Vinka-04 | -0.951 | 0.244 | 30 | -3.90 | 0.0027 |
| Lower internode | Vinka-03 - Vinka-04 | -0.902 | 0.244 | 30 | -3.70 | 0.0045 |
| Middle insertion angle | Vinka-02 - Vinka-03 | -7.775 | 2.544 | 30 | -3.06 | 0.0230 |

**Table S4.** Linear Discriminant Analysis classification accuracy using leave-one-out cross-validation.

| **Variety** | **n** | **Correctly classified** | **Accuracy (%)** |
| --- | --- | --- | --- |
| Vinka-01 | 4 | 3 | 75 |
| Vinka-02 | 10 | 6 | 70 |
| Vinka-03 | 10 | 7 | 80 |
| Vinka-04 | 10 | 10 | 100 |
| **Overall** | **34** | **26** | **82.4** |

.

# Supplementary Figures


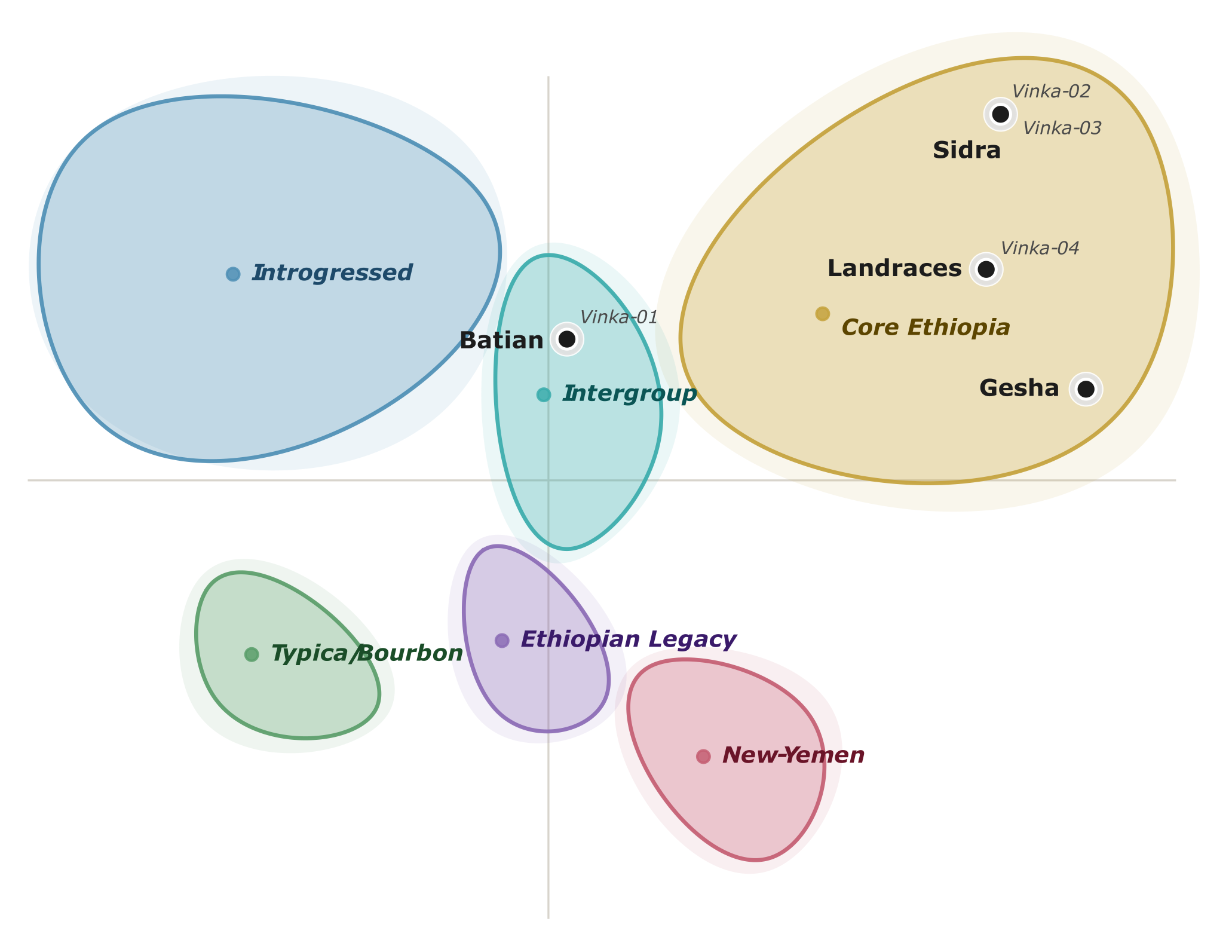


**Figure S1.** Schematic representation of the genetic relationships among *Coffea arabica* morphotypes from VINKA Coffee Farm, southern Ecuador, based on Montagnon et al. (2021) and Montagnon et al. (2025b). Colored areas indicate major genetic groups for *C. arabica* cultivars, and black dots represent the specific cultivar types identified within each group. Vinka-01 clusters as compatible with Batian (Intergroup); Vinka-02 and -03 with Sidra (Core Ethiopia); and Vinka-04 with Ethiopian landraces (Core Ethiopia), genetically close to Gesha (see Table S1). For a comprehensive view of the genetic groups and known C. arabica cultivars, see the Arabica Coffee Cultivars Wheel (RD2 Vision, 2025; <https://rd2vision.com/the-arabica-coffee-cultivars-wheel/>).

**
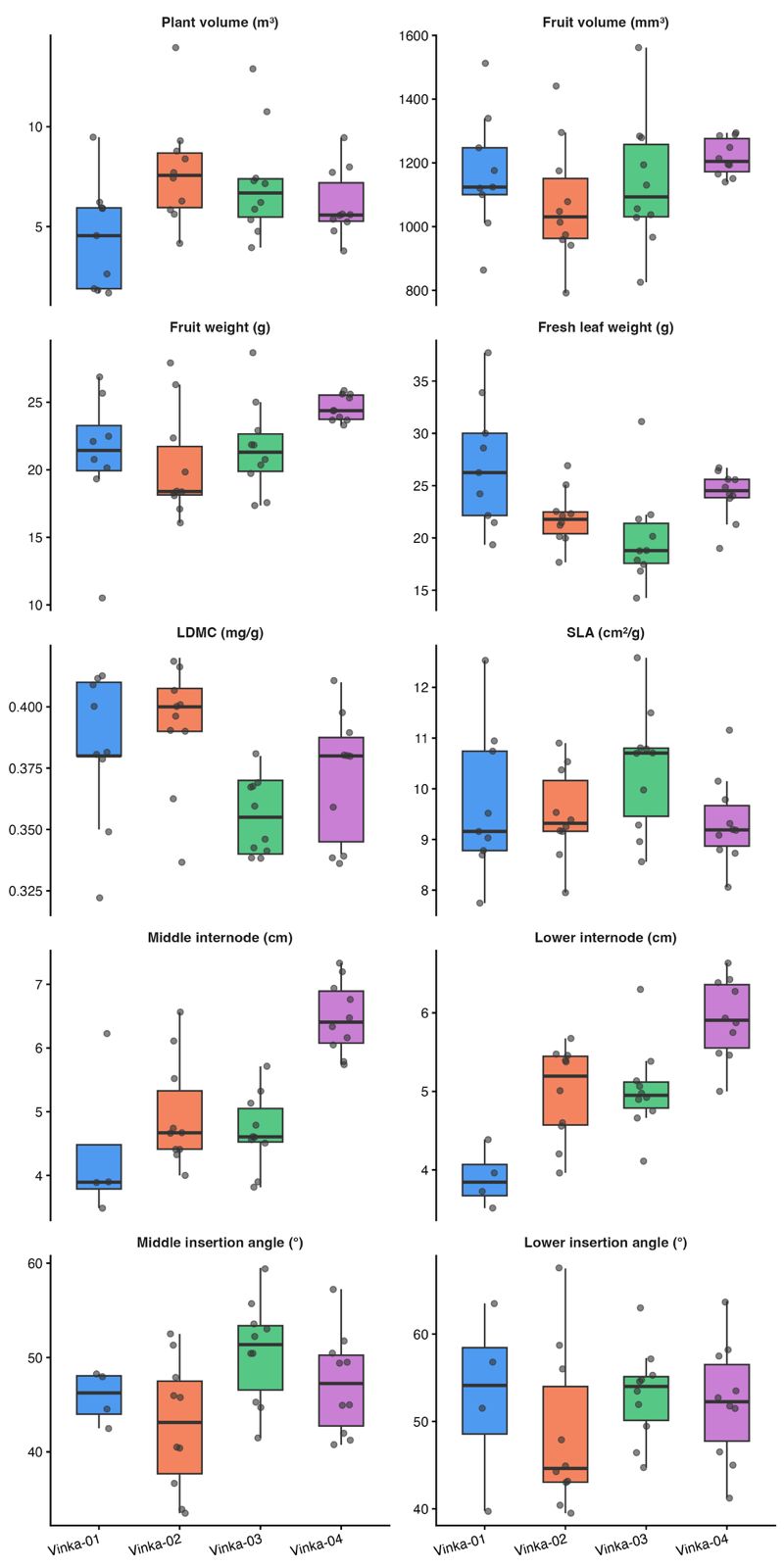
**

**Figure S2.** Morphological trait distributions among *C. arabica* varieties at VINKA Coffee Farm, southern Ecuador. Boxplots show median (center line), interquartile range (box), and range (whiskers) for 10 morphological traits. Individual data points represent measurements from individual plants (Vinka-01: n=9; Vinka-02-4: n=10 each). Traits showing significant differences among varieties (p<0.05): plant volume, fresh leaf weight, LDMC, middle and lower internode length, and middle insertion angle (see Table S2 for statistical tests).
